# Use of Data Mining Techniques for the Prediction of Surface Roughness of Printed Parts in Polylactic Acid (PLA) by Fused Deposition Modeling (FDM): A Practical Application in Frame Glasses Manufacturing

**DOI:** 10.3390/polym12040840

**Published:** 2020-04-06

**Authors:** Esther Molero, Juan Jesús Fernández, Oscar Rodríguez-Alabanda, Guillermo Guerrero-Vaca, Pablo E. Romero

**Affiliations:** Department of Mechanical Engineering, University of Cordoba, Medina Azahara Avenue, 14071 Cordoba, Spain; esther.molero@uco.es (E.M.); p82feruj@uco.es (J.J.F.); orodriguez@uco.es (O.R.-A.); p62rocap@uco.es (P.E.R.)

**Keywords:** fused deposition modeling, FDM, FFF, data mining, machine learning, PLA, surface roughness, WEKA, decision trees, C4.5, neural networks, ANN, frame glasses

## Abstract

In the present work, ten data mining algorithms have been used to generate models capable of predicting the surface roughness of parts printed on polylactic acid (PLA) by using fused deposition modeling (FDM). The models have been trained using experimental data measured on 27 horizontal (XY) and 27 vertical (XZ) specimens, printed using different values for the parameters studied (layer height, extrusion temperature, print speed, print acceleration and flow). The models generated by multilayer perceptron (MLP) and logistic model trees (LMT) have obtained the best results in a cross-validation. Although it does not obtain such optimal results, the J48 algorithm (C4.5) allows the generation of models in the form of a decision tree. These trees permit to determine which print parameters have an influence on the surface roughness. For XY specimens, the surface roughness measured in the direction parallel to the extrusion path (R_a,0,XY_ ) depends on the flow, the print temperature and the layer height; in the direction perpendicular to the extrusion path, the surface roughness (R_a,90,XY_) depends only on the flow. For XZ specimens, the surface roughness measured in the direction parallel to the extrusion path (R_a,0,XZ_) depends only on the print speed; in the direction perpendicular to the extrusion path (R_a,90,XZ_), it depends on the layer height and the extrusion temperature. According to the study carried out, the most suitable set up provides values of R_a,0,XY_, R_a,90,XY_, R_a,0,XZ_ and R_a,90,XZ_ equal to 0.46, 1.18, 0.45 and 11.54, respectively. A practical application of this work is the manufacture of PLA frame glasses using FDM.

## 1. Introduction

Currently, the industry is suffering a profound revolution. Different technological tools are being used intensively in factories [1]: augmented reality, virtual twins, data mining, additive manufacturing, among others. This fact is known as industry 4.0. 

Additive manufacturing (AM) was initially used to manufacture prototypes in the product development stage. Today, it is also used to manufacture: customized objects (rapid prototyping), tools for other processes (rapid tooling), small batches of fully functional parts (rapid manufacturing) [2]. 

There are different technologies of AM. However, fused deposition modeling (FDM) is the most extended technology [3,4,5]. The low cost of the equipment and the diversity of filaments in the market contribute to this. Although there are many materials available, polylactic acid (PLA) is still one of the most widely used in FDM [6,7]. There are several reasons for this [8]: it is biodegradable, easily printed, does not give off any vapors during printing and there are PLA formulas with mechanical properties similar to acrylonitrile butadiene styrene (ABS) [9].

For some applications, 3D printed parts must achieve minimum mechanical properties [8,10,11,12,13]. In other cases, apart from a high tensile strength value, the parts need to have a proper aesthetic quality [14,15,16]. Some authors have evaluated which factors allow achieving the best rankings in all hedonic, tactile and visual assessments of FDM 3D printed parts [17]; in the tactile and visual evaluations, the authors conclude that the pieces with the highest scores are those with the lowest values of surface roughness. On the other hand, the surface finish is crucial when printed parts are in contact with human skin, as is the case with frame glasses [18]. 

One of the motivations for this work is to determine which printing factors are the most influential in the surface finish on PLA parts printed using FDM. In the literature, you can find previous works in which the influence of different printing parameters on the surface roughness of 3D printed parts has been studied (Table 1). However, there are printing parameters that have not yet been analyzed, such as print acceleration or flow. This is one of the main contributions of this work.

Traditionally, the experimental study of the quality of manufactured parts has been carried out with statistical tools, such as the Taguchi method and analysis of variance (ANOVA) [19]. However, the large amount of data currently generated by industry 4.0 sensors and the need to use algorithms capable of modeling non-linear problems has made it necessary to use data mining (also known as machine learning, ML) techniques [20,21,22]. The second motivation for the work is to use data mining (DM) techniques to predict the surface finish of 3D printed parts using FDM. 

Razvi et al. [23] have reviewed the existing literature on the use of DM in additive manufacturing; the papers reviewed have been grouped around four different topics: design, process optimization, monitoring and control, inspection and testing. Amand et al. [24] have used DM techniques to predict possible defects during the configuration step in FDM 3D printing. Wu et al. [25] have used the random forest algorithm to generate a model to predict the surface roughness of a 3D printed part from data collected by different sensors placed in the FDM printer. Sohnius et al. [26] have employed DM techniques to predict the quality of printed parts using FDM from data obtained via the machine vision method. Mahapatra and Sood [27] proposed the use of artificial neural networks (ANN) to determine the relationship between five input FDM parameters such as layer thickness, orientation, raster angle, raster width, and air gap with surface roughness in the top, bottom, and side surface of the acrylic nitrile butadiene styrene (ABS) built part. Boschetto et al. [28] proposed a feed-forward neural network to fit experimental data and to determine surface roughness parameter models reliable over the entire part surface. Vahabli and Rahmati [29] have established a robust model using empirical data based on optimized ANN to estimate the surface roughness distribution in fused deposition modeling ABS parts; this work includes four medical case studies. 

The aim of this work is to generate and validate models via data mining techniques that allow predicting the surface finish of PLA printed parts according to the selected values for the following printing parameters: layer height, extrusion temperature, print speed, print acceleration and flow. For this purpose, 27 horizontal and 27 vertical specimens have been manufactured, according to a fractional experiment design. The surface roughness of these specimens was measured. The results were used to generate and test different models via data mining algorithms (Bayes Net, naïve-Bayes, multilayer perceptron, simple logistics, sequential minimal optimization, IBk, Kstar, J48, logistic model tree and random forest). In addition, using the J48 algorithm, decision trees were generated to determine which printing parameters significantly influence the surface roughness of the parts. 

As a practical application of the work, the manufacture of a frame glasses is proposed. There are standard frames on the market, which are adapted to the characteristics of an average person. However, there are people with such a facial morphology that they cannot find glasses in the optical shops. By means of 3D printing, it is possible to manufacture customized frames [30], in biodegradable materials such as PLA [18]. One of the most important specifications of a frame glasses is the surface finish, for aesthetic reasons and to reduce friction with the skin of the user.

## 2. Materials and Methods

### 2.1. Design of Experiments and Printing of Specimens

**Figure 1:**
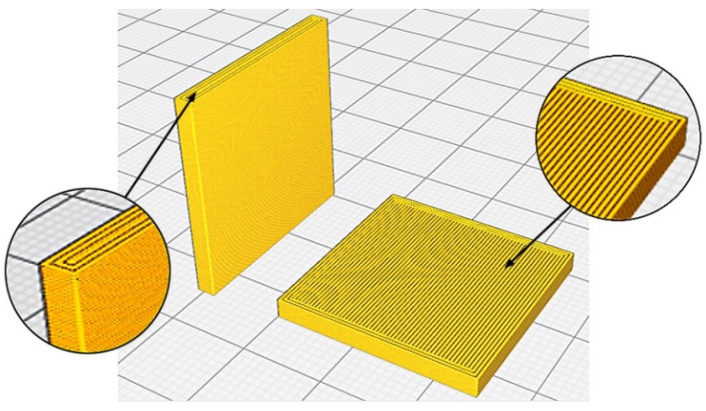
Print orientation: vertical (XZ) (**left**), and horizontal (XY) (**right**).

In this work, 54 test pieces with dimensions 25.0 mm × 25.0 mm × 2.4 mm were printed following a fractional experiment design, with five factors and three levels (Table 2). The factors studied were: layer height (LH), extrusion temperature (T), print speed (PS), print acceleration (PA) and flow (F). Table 3 shows the parameters set in each test. A total of 27 specimens were printed in the XY orientation, and others 27 in the XZ orientation (Figure 1). 

The specimens were designed using SolidWorks (Dassault Systemes, Vélizy-Villacoublay, France). The selection of print parameter values and the generation of the numerical code (CN) was performed using Ultimaker CURA software (version 4.0.0, Ultimaker, Utrecht, The Netherlands).

The specimens were produced on an Ender 3 printer (Creality 3D, Shenzhen, China), with a 220 × 220 × 250 mm^3^ workspace and a hot bed (50 °C). An extrusion nozzle with a diameter of 0.4 mm was used in the tests.

### 2.2. Surface Roughness Measurement

**Figure 2:**
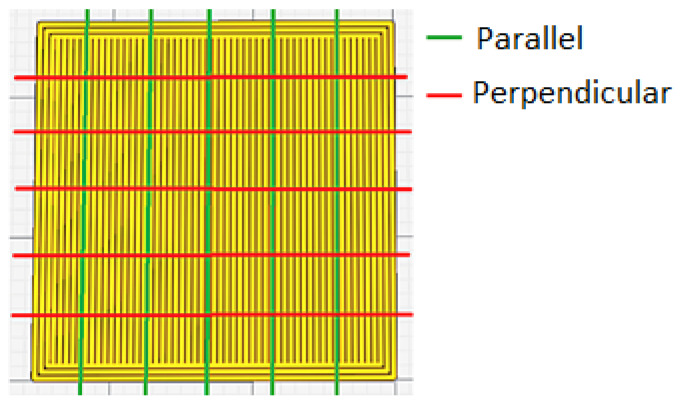
Directions of measurement taken in the roughness test.

Surface roughness (R_a_) of the printed specimens was measured using a Mitutoyo SJ-201 profilometer (Mitutoyo, Kawasaki, Japan). R_a_ was measured five times in the direction parallel to the extrusion path (R_a,0_) and five times in the direction perpendicular to the extrusion path (R_a,90_) (Figure 2). The representative value in each direction for each specimen was calculated as the arithmetic mean of these five measurements. 

### 2.3. Data Mining Algorithms 

Data mining is a discipline that allows us to analyze data sets and extract knowledge from them. It is a key tool in the industry to process the data that is generated daily on machines and manufacturing lines [20].

There are many DM algorithms. These algorithms can be classified into two main groups [35]: supervised and unsupervised. Supervised algorithms are those that work with instances that a priori already belongs to a class. Unsupervised algorithms are used precisely to try to classify instances into groups that were not known a priori. Regression and classification models are the most well-known supervised models while clustering is the main technique of the unsupervised category [36].

The data obtained from the surface roughness measurements have been categorized into two classes, using the median value as the border: surface roughness values below the median value have been categorized as class 1; surface roughness values above the median value have been categorized as class 2. 

From these data, a total of 40 models have been generated, using ten of the most common classification algorithms in data mining. These algorithms are briefly described in Table 4. Two parameters have been used to evaluate each model: the percentage of correctly classified instances and the kappa statistic. These parameters are calculated using the cross-validation procedure (10-folds).

### 2.4. Decision Trees

Decision trees are one of the most widely used supervised algorithms. In this category are [35]: C4.5, CART, random forest, random tree, among others. The C4.5 algorithm was developed by Quinlan [43]. This algorithm generates tree-shaped models that allow the classification of instances in a simple, visual and easy to understand way.

The C4.5 algorithm is based on the concept of entropy, understanding entropy as a measure of data disorder [43]. The entropy of a vector and $\vec{y}$ can be calculated as shown in Equation (1) iterating over all possible values of $\vec{y}$. Conditional entropy is calculated as shown in Equation (2).

(1) $$Entropy\left(\vec{y}\right)=-\overset{n}{\underset{j=1}{\sum }}\frac{\left|y_{j}\right|}{\left|\vec{y}\right|}log\frac{\left|y_{j}\right|}{\left|\vec{y}\right|}$$

(2) $$Entropy\left(j|\vec{y}\right)=\frac{\left|y_{j}\right|}{\left|\vec{y}\right|}log\frac{\left|y_{j}\right|}{\left|\vec{y}\right|}.$$

Finally, the gain is defined as shown in Equation (3).

(3) $$Gain\left(\vec{y},j\right)=Entropy(\vec{y}-Entropy(j|\vec{y})$$

For each node in the tree, the algorithm chooses the attribute that most effectively divides the original set into different smaller subsets. The attribute with the highest information gain is chosen as a decision parameter. The WEKA data mining software, developed by the University of Waikato (Hamilton, New Zealand), includes the J48 algorithm, based on the C4.5 [35]. 

### 2.5. Case Study: Printing a Frame Glasses

As a practical application of the work, the manufacture of a frame glasses is proposed. A frame is made up of two main elements: front and temples. Each of these elements has been personalized for the face/head of one of the authors, who cannot find glasses on the market that fit his morphology. In this case, the front has been printed in a horizontal orientation (XY) and the temples in a vertical orientation (XZ). The printing parameters chosen in each case have been selected after this study.

## 3. Results

### 3.1. Data from the Tests

The mean values and standard deviation of surface roughness in the direction parallel to extrusion path (R_a,0_) and in the direction perpendicular to extrusion path (R_a,90_) for both print orientation (XY and XZ) are shown in Table 5 and Table 6. As expected, due to the deposition of the fused filament layer after layer, the surface roughness in the direction parallel to the extrusion path is lower than the surface roughness in the perpendicular direction. 

### 3.2. Comparison of Models Generated via Data Mining Algorithms

To analyze the data, they have been classified into two groups (Table 7): class 1 (low surface roughness values) and class 2 (high surface roughness values). This data has been processed by the WEKA software. By means of this software, different classification algorithms have been used to predict whether an instance belongs to class 1 or class 2. Each algorithm has been used to generate 4 models: R_a,0XY_, R_a_,_90XY_, R_a_,_0XZ_, R_a,90XZ_. Each model has been evaluated using two criteria: on the one hand, the number of correctly classified instances; on the other, the value of the kappa statistic (Table 8).

**Figure 3:**
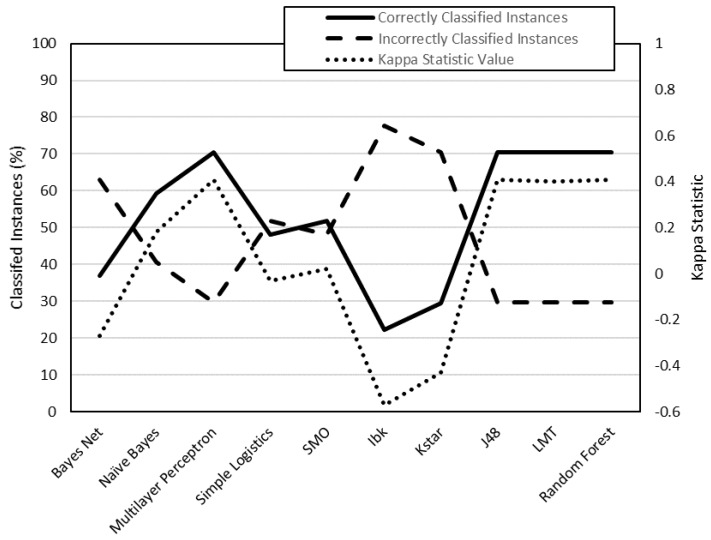
Evaluation of the models generated by WEKA for R_a,0,XY_ data (cross-validation, 10 folds).

**Figure 4:**
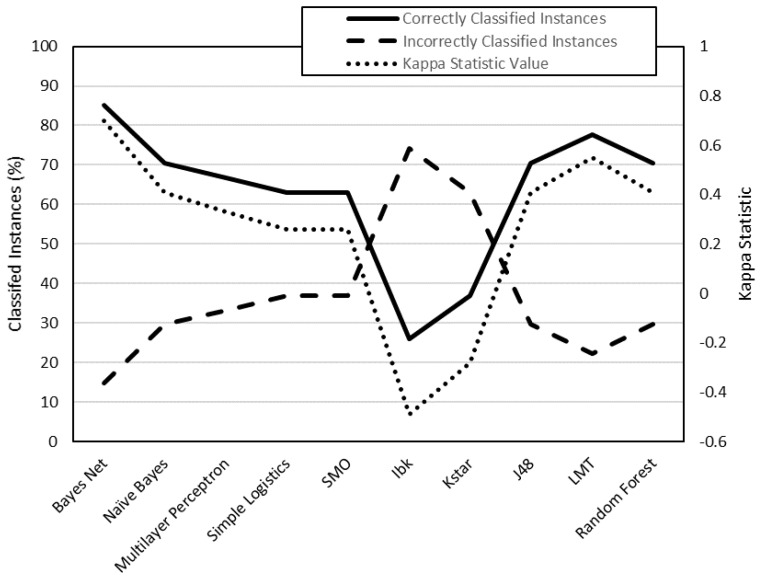
Evaluation of the models generated by WEKA for R_a,90,XY_ data (cross-validation, 10 folds).

**Figure 5:**
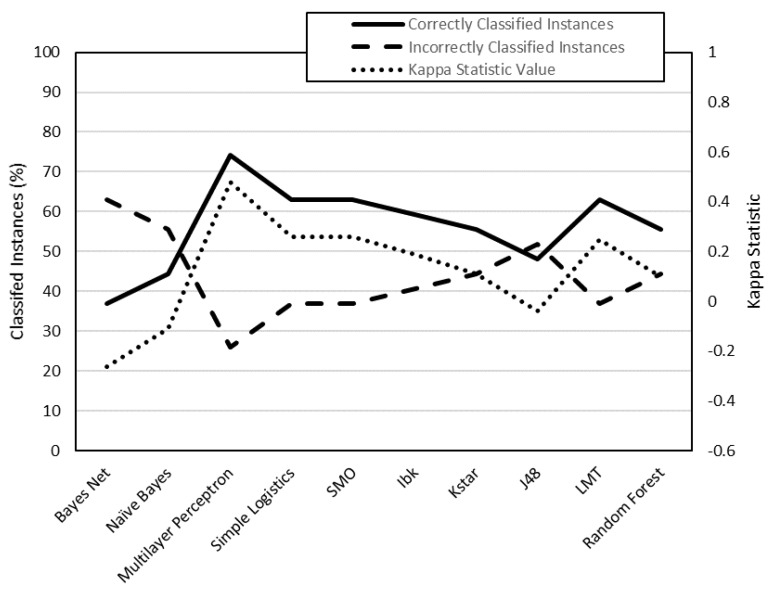
Evaluation of the models generated by WEKA for R_a,0,XZ_ data (cross-validation, 10 folds).

**Figure 6:**
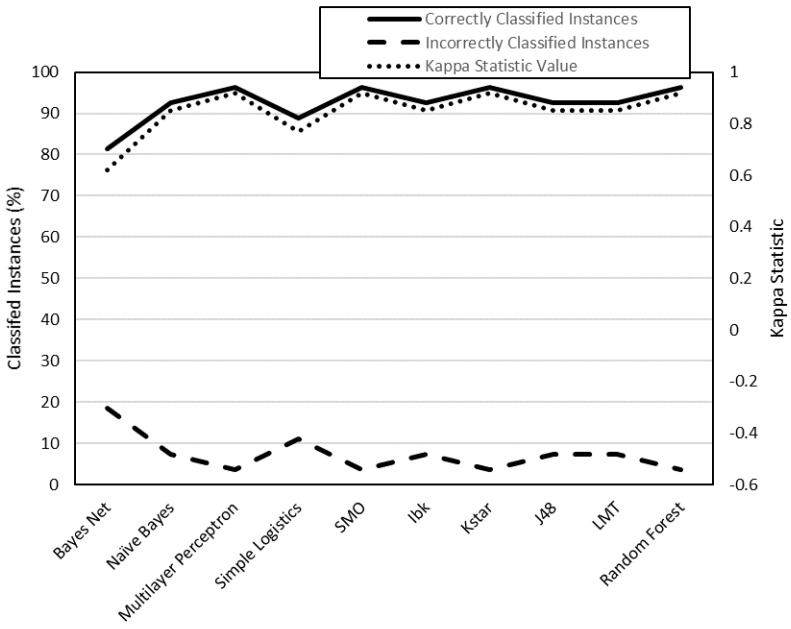
Evaluation of the models generated by WEKA for R_a,9,0XZ_ data (cross-validation, 10 folds).

The models generated from R_a,0,XY_, R_a,90,XY_, R_a0,XZ_ and R_a90,XZ_ data are shown in Figure 3, Figure 4, Figure 5 and Figure 6, respectively. The algorithms that achieve better results for Ra_0,XY_ are MLP, J48, LMT and random forest. The algorithms that achieve better for Ra_90,XY_ results are Bayes Net and LMT. The algorithm that achieves better results for Ra_0,XZ_ are MLP and LMT. The algorithms that achieve better results for Ra_90,XZ_ are MLP, SMO, Kstar and random forest.

### 3.3. Generation of Decision Trees (J48)

As seen in the previous section, except in the case of Ra_0,XZ_, the models generated by the J48 algorithm present adequate results. On the other hand, this algorithm is not a black box: it allows the generation of decision trees that are easy to interpret and that provide additional information.

**Figure 7:**
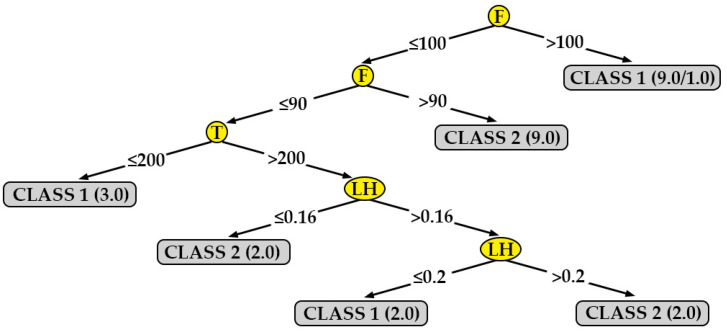
Decision tree for surface roughness measured in the direction parallel to the extrusion path on printed specimens in the XY orientation (R_a,0,XY_).

Figure 7 shows the decision tree generated for the surface roughness in the direction parallel to the extrusion path for the data obtained from specimens printed in the XY orientation (R_a,0,XY_). As it can be seen in the figure, the most important parameters are flow (F), extrusion temperature (T) and layer height (LH). According to this tree, a lower surface roughness (class 1) can be obtained in the following cases:

- By selecting a flow value (F) higher than 100 % (directly).
- By selecting a flow value (F) equal to 90 % and a print temperature (T) equal to 200 °C.
- By selecting a flow (F) value equal to 90 %, a print temperature (T) higher than 200 °C and a layer height (LH) equal to 0.2 mm.

**Figure 8:**
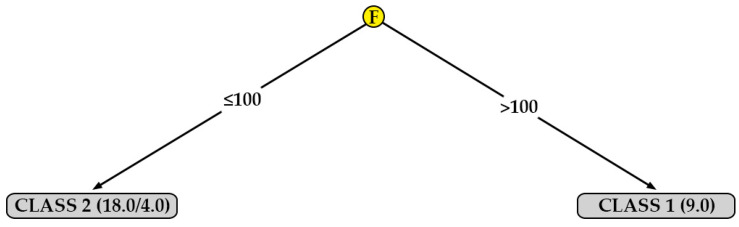
Decision tree for surface roughness measured in the direction perpendicular to the extrusion path on printed specimens in the XY orientation (R_a,90,XY_).

Figure 8 shows the decision tree generated for the surface roughness in the direction perpendicular to the extrusion path, for the data obtained from specimens printed in XY orientation (R_a,90,XY_). In this case, the most important parameter is the flow (F). According to this tree, a lower surface roughness (class 1) can be obtained simply by selecting a flow value (F) higher than 100%.

**Figure 9:**
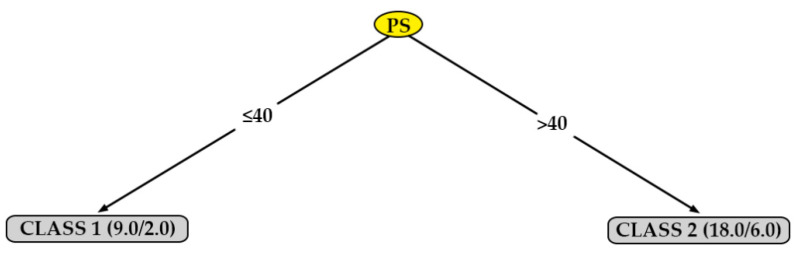
Decision tree for surface roughness measured in the direction parallel to the extrusion path on printed specimens in the XZ orientation (R_a,0,XZ_).

Figure 9 shows the decision tree generated for surface roughness in the direction parallel to the extrusion path from the data obtained from specimens printed in XZ position (R_a,0,XZ_). As can be seen in this figure, the most important parameter, in this case, is the print speed (PS). According to this tree, a lower surface roughness (class 1) can be obtained simply by selecting a value for the printing speed (PS) equal to 40 mm/s.

**Figure 10:**
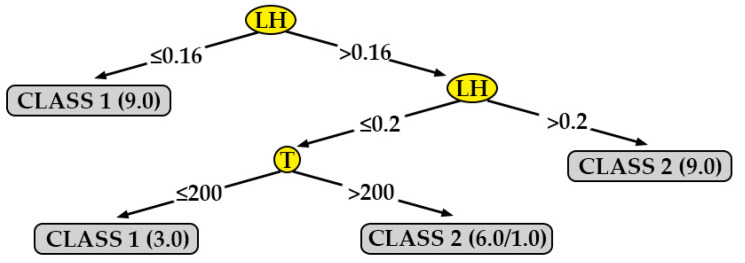
Decision tree for surface roughness measured in the direction perpendicular to the extrusion path on printed specimens in the XZ orientation (R_a,90,XZ_).

Figure 10 shows the decision tree generated for roughness in the direction perpendicular to the extrusion path, for the data obtained from specimens printed in XZ position (R_a,90,XZ_). In this case, the most important parameters are the layer height (LH) and the extrusion temperature (T). According to this tree, a lower surface roughness (class 1) can be obtained in the following cases:

- By selecting a layer height (LH) equal to 0.16 mm (directly).
- By selecting a layer height (LH) equal to 0.20 mm and a print temperature (T) equal to 200 °C.

Table 9 shows the percentages of correctly and incorrectly classified instances, as well as the kappa statistic for each model. According to Table 8, the models generated for R_a,0,XY_ and R_a,90,XY_ is rated as ‘moderate’; on the other hand, the model generated for R_a,90,XZ_ is rated as ‘almost perfect’. Finally, the model obtained for R_a,0,XZ_ obtains very poor results. The results obtained from the first three models are supported. This is not the case with the last model.

**Figure 11:**
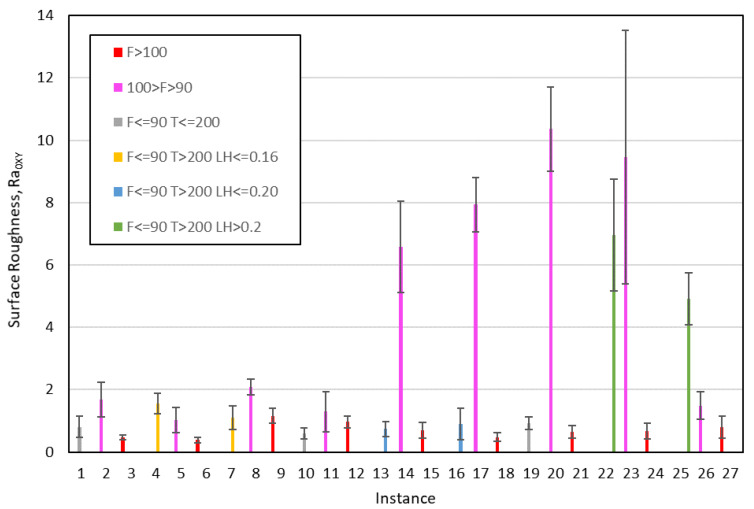
Mean values and standard deviation for R_a0XY_; each color represents a leaf of the decision tree: pink, yellow and green instances belong to class 2; the rest of color belong to class 1 (F: Flow; T: Temperature; LH: layer height).

**Figure 12:**
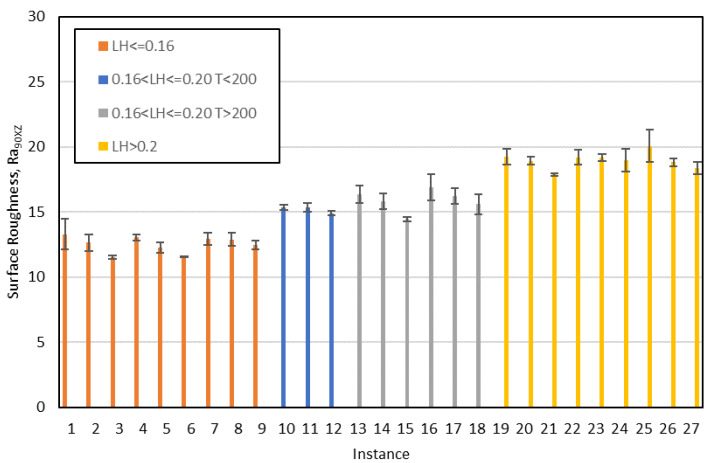
Mean values and standard deviation for R_a90XZ_; each color represents a leaf of the decision tree: orange and blue instances belong to class 1; grey and yellow ones belong to class 2 (LH: layer height; T: temperature).

In order to check that the information extracted from the decision trees is correct, the mean value and the standard deviation obtained for surface roughness in each of the 27 tests carried out have been represented. As an example, Figure 11 and Figure 12 show the results for R_a0XY_ and R_a90XZ_, respectively. Figure 11 clearly shows the instances belonging to class 1 (F > 100; F ≤ 90 and T ≤ 200; F ≤ 90 and T > 200 and LH = 0.2) and the instances belonging to class 2 (the rest). Likewise, Figure 12 shows that the instances that meet certain criteria (LH ≤ 0.16; 0.16 < LH ≤ 0.20 and T < 200) have a lower surface roughness than the rest.

### 3.4. Practical Application: Reduction of Surface Roughness in FDM 3D Printed Frame Glasses

**Figure 13:**
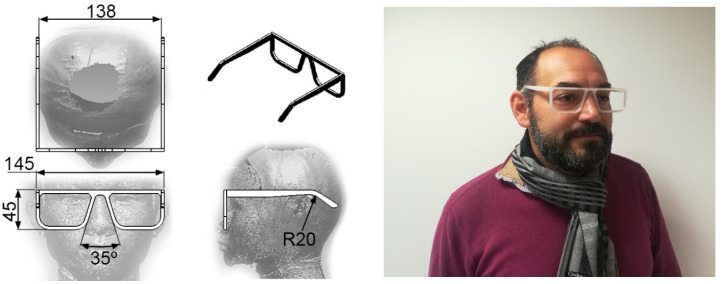
Customized frame glasses: parameterized distances (**left**); real frame glasses produced in PLA via FDM (F = 110%; LH = 0.16 mm) (**right**).

A practical application of the results obtained in this work is the production of frame glasses printed in PLA using FDM (Figure 7). Frames glasses are a perfect example of a product that should be custom-made: each user has a face width, a nose width and a face-to-ear distance (Figure 13, left). The most important dimensions of a frame glasses can be parameterized in a CAD design and adjusted according to the needs of each customer. Once the model was customized, it was printed using an F equal to 110% and an LH equal to 0.16 mm (Figure 13, right). 

## 4. Discussion

In the present work, data mining techniques have been used to generate models that allow predicting the surface roughness of parts printed in PLA using 3D FDM printing. The classifier algorithms used to generate the models have been: Bayes net, naïve-Bayes, multilayer perceptron (ANN), simple logistic, SMO (SVM), IBk (KNN), KStar, J48 (C4.5), LMT y random forest. The models have been tested by means of a cross-validation (10-folds). The percentage of correctly classified instances and the kappa statistic were used to compare these models with each other. The input variables of the study have been: print orientation, layer height (LH), extrusion temperature (T), print speed (PS), print acceleration (PA) and flow (F). The output variables have been the surface roughness measured in the direction parallel to the extrusion path (R_a,0_) and in the direction perpendicular to the extrusion path (R_a,90_). A total of 54 specimens have been printed for this purpose: 27 specimens were printed with horizontal orientation (XY) and the other 27 with vertical orientation (XZ). 

From the results shown in Figure 3, Figure 4, Figure 5 and Figure 6, it can be stated that one of the models that best classify the instances according to their surface roughness (both for horizontal and vertical specimens) is the one generated by the multilayer perceptron (artificial neural network, ANN) algorithm. In the literature there are works that also use different types of ANN to predict the surface roughness of printed parts using FDM: Boschetto et al. [28] propose the use of a feed-forward neural network to predict surface roughness on surfaces that form different angles to the vertical, obtaining errors of less than 5%; Vahabli and Rahmati [29] propose a similar model, using another type of ANN, capable of predicting surface roughness as a function of build angle. However, both works do not take into account the influence of printing parameters on the surface roughness obtained.

Another algorithm that has generated in the present work models with positive results has been the LMT. This fact is consistent with other works of literature: Landwehr et al. [46] concluded that LMT produces more accurate classifiers than J48 (C4.5), CART, logistic regression, models tree, functional trees, naïve Bayes trees and LOTUS. The problem with this algorithm, like ANN, is that it does not generate a model that can be visualized or easily understood.

One algorithm that generates a model that can be represented graphically is J48. Figure 7, Figure 8, Figure 9 and Figure 10 show the decision trees generated by this algorithm for the different measured surface roughness. From the models obtained, Table 10 has been drawn up. This table summarises the printing variables that must be taken into account in order to obtain a lower surface roughness (class 1). From these decision trees, a basic configuration can be established to obtain a good surface finish simultaneously on XY and XZ orientation, for perpendicular and parallel direction to extrusion path: F equal to 110 % and LH equal to 0.16 mm.

In addition to the above, the following statements can be made:

- The decision tree models can be easily interpreted by any 3D printer operator (they are not black-box models) [47]. For this reason, they are used for modeling other manufacturing processes in literature [48]. This is important in the current industry that is very concerned with visual management.
- Two parameters that had not been previously studied in the literature on PLA specimens were included in this work: print acceleration and flow. While print acceleration seems not to have an influence in any of the cases studied, the flow is revealed as an important parameter in the surface roughness obtained in XY orientation. This result is consistent with those obtained by the authors in similar tests performed on PETG [47].
- In R_a,90,XZ_, the most influential parameters are layer height. These results coincide with those obtained by other authors: García-Plaza et al. [31] state that the layer height parameter is the most influential in upright and on-edge positions (similar to the XZ orientation in the present paper).
- Three of the four models generated have achieved a ’moderate’ or ’almost perfect’ rating, according to their kappa statistic values. This fact supports the validity of the results obtained in the present work, as shown in Figure 11 and Figure 12. The model generated for R_a0,XZ_ did not pass the evaluation.
- The results obtained in the present work have been used to print a frame glasses in PLA via FDM. A-frame glasses is the perfect example of a customized part/assembly. There are previous works in the literature focused on this topic [18], although they have not studied the printing parameters that allow to achieve a better surface finish.

## 5. Conclusions

In the present work, data mining algorithms have been used to generate from experimental tests models capable of predicting the surface roughness of horizontal and vertical parts printed in PLA using FDM. MLP and LMT are the algorithms that obtain the best results in cross validation tests.

The J48 algorithm obtains almost perfect results in one of the models and moderate results in two others. However, unlike MLP and LMT, this algorithm generates decision trees in which it is possible to see which print parameters influence surface roughness. 

Of the five parameters studied, two have proved to be the most important for obtaining a better surface finish in XY and XZ specimens: flow and layer height. A flow equal to 110% and a layer height equal to 0.16 mm provides the lowest values of surface roughness (R_a_) in PLA printed specimens in XY and XZ orientation. Using these values, a frame of glasses has been printed, as a practical and direct application of this work.

## Figures and Tables

**Table 1:** Previous works that have studied the surface roughness of polylactic acid (PLA) printed parts using fused deposition modeling (FDM).

Author	Layer Height	Tempe-Rature	Print Orienta-tion	Print Speed	Filling Density	Nozzle Diame-ter	Wall Thick-ness	Print Path
García-Plaza et al. [31]	√	-	√	√	-	-	-	-
Ramli et al. [32]	√	-	-	-	√	-	-	-
Alsoufi and Elsayed [33]	√	-	-	-	-	√	-	-
Kovan et al. [34]	√	√	-	-	-	-	-	-
Perez et al. [19]	√	√	-	√	-	-	√	√

**Table 2:** Factors and levels used in the design of experiments (DOE).

Factors	Level 1	Level 2	Level 3
Layer height, LH (mm)	0.16	0.20	0.24
Temperature, T (°C)	200	210	220
Print speed, PS (mm/s)	40	50	60
Print acceleration, PA (mm/s^2^)	500	1000	1500
Flow, F (%)	90	100	110

**Table 3:** Design of experiment L27 used in the present work.

Layer Height, LH (mm)	Temperature, T (°C)	Print Speed, PS (mm/s)	Print Acceleration, PA (mm/s^2^)	Flow, F (%)
0.16	200	40	500	900
0.16	200	40	500	100
0.16	200	40	500	110
0.16	210	50	1000	90
0.16	210	50	1000	100
0.16	210	50	1000	110
0.16	220	60	1500	90
0.16	220	60	1500	100
0.16	220	60	1500	110
0.20	200	50	1500	90
0.20	200	50	1500	100
0.20	200	50	1500	110
0.20	210	60	500	90
0.20	210	60	500	100
0.20	210	60	500	110
0.20	220	40	1000	90
0.20	220	40	1000	100
0.20	220	40	1000	110
0.24	200	60	1000	90
0.24	200	60	1000	100
0.24	200	60	1000	110
0.24	210	40	1500	90
0.24	210	40	1500	100
0.24	210	40	1500	110
0.24	220	50	500	90
0.24	220	50	500	100
0.24	220	50	500	110

**Table 4:** Description of the data mining algorithms used in the present work (elaborated from [37]).

Algorithm	Description	Ref.
Bayes Net (BN)	It is a Bayesian classification algorithm that provides joint conditional probability distributions. BN algorithm consists of a directed acyclic graph and a set of conditional probability tables. Each random variable is expressed by a node in the directed acyclic graph. The conditional probability table for the values of the variables indicate each possible combination of the values of its parent nodes.	[38]
Naïve-Bayes	It is a statistical classification algorithm which is based on Bayes’ theorem. The suppositions of accepting that predictive attributes are conditionally independent given the class and no hidden or latent attributes influence the predictive process make the algorithm a suitable tool for classification and learning.	[39]
Multilayer Perceptron (MLP)	MLP is a feed-forward neural network with one or more hidden layers that uses back-propagation to classify instances. The structure of an MLP typically consists of an input layer, hidden layers and output layer, where the input signals are propagated in the forward direction.	[40]
Simple Logistics	Logistic regression is a statistical model that predicts the probability of some event occurring as a linear function of a set of predictor variables. Linear regression presents two problems: the membership values are not proper probability values and the least-squares regression takes errors as both statistically independent and normally distributed with the same standard deviation. In order to get rid of these problems, logistic regression generates a linear model based on a transformed target variable.	[35]
Sequential Minimal Optimization (SMO)	It is a support vector machine (SVM) classifier that employs sequential minimal optimization for training. SVM is a method for classification of linear and nonlinear data that uses a nonlinear mapping for transforming the original data into a higher dimension.	[38]
IBk	It is an instance-based learning algorithm which is a slightly modified version of the K-nearest neighbor (KNN) algorithm. The algorithm can determine the appropriate value for K based on cross-validation. It normalizes the ranges of attributes, processes instances incrementally and has a policy for tolerating missing values.	[41]
KStar	It is an instance-based learning algorithm which uses an entropy-based distance function. It handles with symbolic attributes, real-valued attributes and missing values properly owing to the use of entropy as a distance function. The technique of summing probabilities over all possible paths overcomes the problem of smoothness.	[42]
J48	J48 is a slightly modified version of C4.5 in WEKA. C4.5 is a successor of ID3 algorithm. The test attribute selection criteria of the algorithm is information gain to overcome the attribute bias problem of ID3. For a given set, each time the algorithm selects an attribute with the highest information gain.	[43]
Logistic Model Trees (LMT)	LMT is a classification algorithm that integrates decision tree induction with logistic regression. The tree structure of the algorithm is grown in a similar manner to the C4.5 algorithm. Here, an iterative training of additive logistic regression models is performed. By splitting, the logistic regressions of the parent node are passed to the child nodes. This provides to have all parent models and probability estimates for each class at the leaf nodes of the final model.	[44]
Random Forest	It is an ensemble of classification or regression trees, induced from bootstrap samples of the training data. In this model, the generalization error of the classifier depends on the power of the individual trees and the association between the trees. Random feature selection is used in the tree induction process. This enables the algorithm to perform comparable to the Adaboost algorithm and to be tolerable with noisy data.	[45]

**Table 5:** Mean and standard deviation of surface roughness for XY specimens.

Test	R_a,0,XY_ (µm)		R_a,90,XY_ (µm)	
	Mean	Std. Dev.	Mean	Std. Dev.
1	0.80	0.34	0.71	0.34
2	1.68	0.56	1.41	0.75
3	0.46	0.08	1.18	1.59
4	1.55	0.33	1.96	1.87
5	1.02	0.41	2.14	2.49
6	0.39	0.09	2.16	3.33
7	1.11	0.38	2.83	3.63
8	2.09	0.25	3.45	4.05
9	1.16	0.23	3.46	4.82
10	0.59	0.17	3.59	5.56
11	1.29	0.64	4.31	5.80
12	0.96	0.18	4.38	6.61
13	0.74	0.24	4.66	7.23
14	6.58	1.47	7.35	6.30
15	0.69	0.26	5.32	8.39
16	0.89	0.50	5.80	8.84
17	7.93	0.86	8.60	8.09
18	0.48	0.15	6.21	10.21
19	0.92	0.20	6.70	10.65
20	10.36	1.35	10.57	9.33
21	0.64	0.20	7.28	11.88
22	6.96	1.79	10.25	10.50
23	9.46	4.06	12.17	9.76
24	0.67	0.25	8.31	13.59
25	4.91	0.83	10.25	12.94
26	1.48	0.44	9.31	14.47
27	0.80	0.36	9.39	15.26

**Table 6:** Mean and standard deviation of surface roughness for XZ specimens.

Test	R_a,0,XZ_ (µm)		R_a,90,XZ_ (µm)	
	Mean	Std. Dev.	Mean	Std. Dev.
1	0.58	0.25	13.29	1.15
2	0.57	0.21	12.63	0.66
3	0.45	0.03	11.54	0.13
4	0.39	0.13	13.04	0.25
5	0.40	0.10	12.25	0.42
6	0.60	0.23	11.55	0.05
7	0.48	0.15	12.92	0.49
8	0.66	0.11	12.87	0.51
9	1.12	1.11	12.44	0.35
10	0.76	0.24	15.31	0.20
11	0.37	0.08	15.34	0.32
12	0.34	0.10	14.89	0.16
13	0.69	0.55	16.35	0.68
14	0.71	0.16	15.81	0.61
15	0.65	0.16	14.44	0.14
16	0.63	0.18	16.91	1.00
17	0.34	0.12	16.23	0.62
18	0.42	0.06	15.58	0.77
19	0.67	0.20	19.25	0.59
20	0.56	0.25	18.93	0.33
21	0.72	0.30	17.86	0.10
22	0.58	0.27	19.19	0.55
23	0.63	0.18	19.16	0.28
24	0.46	0.16	18.94	0.88
25	0.67	0.19	20.06	1.25
26	0.74	0.25	18.80	0.33
27	0.64	0.21	18.36	0.50

**Table 7:** Values used to establish the classes needed to perform data processing at WEKA.

Orientation	Direction	Class 1	Class 2
XY	0°	0.39–1.02 µm	1.03–10.36 µm
XY	90°	5.79–13.43 µm	13.44–18.69 µm
XZ	0°	0.34–0.60 µm	0.61–1.12 µm
XZ	90°	11.54–15.58 µm	15.59–20.06 µm

**Table 8:** Strenght of concordance for kappa statistic.

Kappa Statistic	Strength of Concordance
0.00	Poor
0.01–0.20	Slight
0.21–0.40	Fair
0.41–0.60	Moderate
0.61–0.80	Substancial
0.81–1.00	Almost perfect

**Table 9:** Information regarding decision tree models generated in this work.

Tree	Correctly Classified Instances (%)	Incorrectly Classified Instances (%)	Kappa Statistics
R_a,0,XY_	70.37	29.63	0.41
R_a,90,XY_	70.37	29.63	0.41
R_a,0,XZ_	44.44	55.56	−0.11
R_a,90,XZ_	92.59	7.41	0.85

**Table 10:** Parameters involved in each data tree models.

	XY	XZ
R_a,0_	**F**, T, LH	PS
R_a,90_	**F**	**LH**, T

NOTE: The most important parameters have been highlighted in bold.
